# Facile Preparative Access to Bioactive Silicon Oxycarbides with Tunable Porosity

**DOI:** 10.3390/ma12233862

**Published:** 2019-11-22

**Authors:** Fangtong Xie, Emanuel Ionescu, Marcela Arango-Ospina, Ralf Riedel, Aldo R. Boccaccini, Isabel Gonzalo-Juan

**Affiliations:** 1 Institute of Materials Science, Technische Universität Darmstadt, Otto-Berndt-Str. 3, D-64287 Darmstadt, Germany; xie@materials.tu-darmstadt.de (F.X.); riedel@materials.tu-darmstadt.de (R.R.); gonzalo@materials.tu-darmstadt.de (I.G.-J.); 2 Institute of Biomaterials, University of Erlangen-Nuremberg, Cauerstrasse 6, D-91058 Erlangen, Germany; marcela.arango@fau.de (M.A.-O.); aldo.boccaccini@ww.uni-erlangen.de (A.R.B.)

**Keywords:** sol-gel, silicon oxycarbide, mesoporosity, specific surface area, apatite forming ability, bioactivity

## Abstract

In the present work, Ca-containing silicon oxycarbides (SiCaOC) with varying Ca content have been synthesized via sol-gel processing and thermal treatment in inert gas atmosphere (pyrolysis). It has been shown that the as-prepared SiCaOC materials with low Ca loadings (Ca/Si molar ratios = 0.05 or 0.12) were X-ray amorphous; their glassy network contains Q^3^ sites, indicating the presence of Ca^2+^ at non-bridging-oxygen sites. SiCaOC with high Ca content (i.e., Ca/Si molar ratio = 0.50) exhibits the presence of crystalline calcium silicate (mainly pseudowollastonite). Furthermore, it has been shown that the incorporation of Ca into the SiOC glassy network has a significant effect on its porosity and specific surface area. Thus, the as-prepared Ca-free SiOC material is shown to be non-porous and having a specific surface area (SSA) of 22.5 m^2^/g; whereas SiCaOC with Ca/Si molar ratio of 0.05 exhibits mesoporosity and a SSA value of 123.4 m^2^/g. The further increase of Ca content leads to a decrease of the SSA and the generation of macroporosity in SiCaOC; thus, SiCaOC with Ca/Si molar ratio of 0.12 is macroporous and exhibits a SSA value of 39.5 m^2^/g. Bioactivity assessment in simulated body fluid (SBF) confirms the hydroxyapatite formation on all SiCaOC samples after seven days soaking, unlike the relatively inert ternary silicon oxycarbide reference. In particular, SiCaOC with a Ca/Si molar ratio of 0.05 shows an increased apatite forming ability compared to that of SiCaOC with Ca/Si molar ratio of 0.12; this difference is considered to be a direct consequence of the significantly higher SSA of the sample with the Ca/Si ratio of 0.05. The present work indicates two effects of Ca incorporation into the silicon oxycarbide glassy network on its bioactivity: Firstly, Ca^2+^ is shown to contribute to the slight depolymerization of the network, which clearly triggers the hydroxyapatite formation (compare the bioactive behavior of SiOC to that of SiCaOC with Ca/Si molar ratio 0.12 upon SBF exposure); secondly, the Ca^2+^ incorporation seems to strongly affect the porosity and SSA in the prepared SiCaOC materials. There is an optimum of Ca loading into the silicon oxycarbide glassy network (at a Ca/Si molar ration of 0.05), which provides mesoporosity and reaches maximum SSA, both highly beneficial for the bioactive behavior of the materials. An increase of the Ca loading leads, in addition to the crystallization of calcium silicates, to a coarsening of the pores (i.e., macroporosity) and a significant decrease of the SSA, both negatively affecting the bioactivity.

## 1. Introduction

Silicon oxycarbide (SiOC) has high biocompatibility [1,2,3] and shows bioactivity, i.e., apatite forming ability upon in vitro simulated body fluid (SBF) exposure [4,5]. By introducing alkaline earth metals such as Ca, Mg into SiOC, its bioactivity can be improved significantly [6,7]. Thus, bioactive Ca-containing SiOC may be suitable substitutes for alkali-containing bioactive silicate glasses, since high alkali dissolution and the related fast pH increase have been reported to induce certain cytotoxicity [8,9,10]. Furthermore, for potential bone regeneration application, materials with defined porosity are beneficial, since, for example, mesopores can be utilized for the delivery of growth factors or antibiotics [3,11].

Metal-containing silicon oxycarbides have been synthesized via the so-called polymer-derived ceramic method (PDC): The incorporation of metal ions into silicon oxycarbide occurs by modifying its polymer precursors, such as polysiloxanes or polysilsesquioxanes, with suitable metal organic compounds [12,13]. Highly porous PDC derived SiOC can be achieved by choosing sol-gel synthesized precursors [3,14], using mesoporous templates [15], or applying post-synthesis treatments of HF etching or air calcination [16]. Particularly, the versatility of the sol-gel approach is promising for preparing SiOC with tunable porosity, and the achieved porous SiOC materials were reported to possess high specific surface areas (i.e., > 500 m^2^/g) [14,17,18].

Typically, sol-gel derived metal-containing silicon oxycarbides are realized via co-polymerization between alkoxysilanes and metal alkoxides [19,20]. However, the use of metal alkoxides requires the usage of (possibly toxic) organic solvents, which is not recommendable from environmental aspects. Furthermore, metal alkoxides, such as calcium alkoxides, are costly and difficult to handle due to their high tendency to hydrolyze with moisture in air [21]. Therefore, a clean (water-based) process to prepare metal-containing silicon oxycarbides is necessary, especially for bioactive applications, as it has been used for the synthesis of silicate-based bioactive [22,23]. The investigation of the feasibility of such inorganic metal source (calcium nitrate) for modifying silicon oxycarbides, and its influence on the material porosity, will be the focus of the present study.

For this purpose, Ca-containing silicon oxycarbides (SiCaOC) were thermally converted from xerogels derived from sol-gel synthesis of triethoxymethylsilane and calcium nitrate in inert gas atmosphere. The porous structure and specific surface area of prepared silicon oxycarbide samples were analyzed with N_2_ sorption method, accompanied with SEM surface morphology analysis. A correlation between the final porosity of silicon oxycarbide samples and the influence of calcium nitrate loading during sol-gel process has been discussed. Furthermore, in vitro SBF bioactivity assessment has been conducted, in order to investigate the effects of Ca loading and porosity on bioactive behavior, i.e., apatite forming ability.

## 2. Experimental Procedure 

### 2.1. Materials Preparation

**Figure 1:**
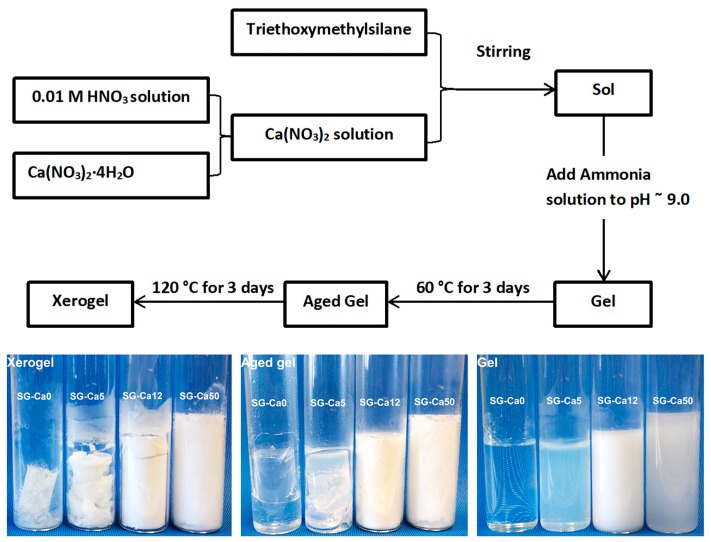
Sol-gel procedure to prepare silicon oxycarbide xerogel precursors with optical pictures of gels, aged gels, and xerogels.

Triethoxymethylsilane (CH_3_Si(OC_2_H_5_)_3_, Alfa Aesar, Kandel, Germany) and calcium nitrate tetrahydrate (Ca(NO_3_)_2_·4H_2_O, Carl Roth, Karlsruhe, Germany) were mixed with an aqueous solution of nitric acid (0.01 M) and stirred until a homogeneous sol was formed. The pH value of the sol was adjusted to 9.0 by adding ammonia solution (1 M), and the gelation process proceeded. The resulting gel was aged at 60 °C and subsequently dried at 120 °C (Figure 1). By varying the Ca/Si molar ratio (0.00, 0.05, 0.12, and 0.50, in which Ca/Si = 0.00 refers to sol-gel synthesis without calcium nitrate addition), four xerogels were prepared and have been denoted as SG-Ca0, SG-Ca5, SG-Ca12, and SG-Ca50, respectively (Table 1).

The xerogel samples were ground to powder and then pyrolyzed at 1100 °C in argon flow to silicon oxycarbide materials. During the heating stage, a holding time of 2 h at 600 °C was conducted, in order to eliminate the residual nitrate groups of calcium nitrate, which were reported to decompose at 500–700 °C [22]. The achieved silicon oxycarbide materials were ground for further analysis, and the powder samples derived from SG-Ca0, SG-Ca5, SG-Ca12, and SG-Ca50 were denoted as SiCa0, SiCa5, SiCa12, and SiCa50, respectively.

### 2.2. Simulated Body Fluid Assessment

In vitro acellular bioactivity evaluation was performed on pyrolyzed silicon oxycarbide samples upon exposure to a simulated body fluid (SBF). The SBF was prepared according to Kokubo et al. [24], and the test was conducted according to the TC04 method (Technical Committee on Bioglasses) reported by Macon et al. [25]. During SBF test, the solid powders were mixed with SBF solution in a solid-liquid ratio of 75 mg to 50 mL in airtight poly-ethylene (PE) bottles. The mixtures were then incubated at 37 °C in a dryer for 7 days. After the incubation, the solid powders were separated from the liquid via filtration and subsequently washed with deionized water and acetone before drying at 40 °C. The obtained SBF-tested powders of SiCa0, SiCa5, SiCa12, and SiCa50 have been denoted as SiCa0-T, SiCa5-T, SiCa12-T, and SiCa50-T, respectively.

### 2.3. Materials Characterization

The prepared xerogel, silicon oxycarbide, and SBF-tested silicon oxycarbide powders were analyzed with X-ray diffraction and FTIR spectroscopy. For X-ray diffraction, a transmission diffractometer (STOE STADI P, Darmstadt, Germany) equipped with Mo Kα X-ray source, Ge(111) monochromator and linear position sensitive detector (PSD) was used to measure diffractograms in a 2Ө range from 5° to 45°. For FTIR spectroscopy, powder samples were mixed with KBr in a concentration of 0.2–1 wt.% (0.2 wt.% for xerogels and 1 wt.% for silicon oxycarbide), and then pressed into transparent or translucent discs with 12 mm diameter. Transmittance spectra of the prepared discs were then collected on a Varian IR-670 spectrometer (Agilent, Santa Clara, CA, USA).

Carbon and oxygen content in SiCa0, SiCa5, SiCa12, and SiCa50 were determined by elemental analysis with a carbon analyzer Leco-200 (Leco Corporation, St. Joseph, CA, USA) and a nitrogen/oxygen analyzer Leco TC-436 (Leco Corporation, St. Joseph, CA, USA), respectively. The surface morphology of silicon oxycarbide samples before and after SBF test was investigated with scanning electron microscopy. Before the measurement, sample powders were distributed on conductive carbon pads and subsequently sputtered with gold. The coated samples were studied on a JEOL JSM-7600F field emission scanning electron microscope (JEOL, Tokyo, Japan).

Nitrogen sorption analysis of xerogel and silicon oxycarbide samples was conducted with QuantaChrome Autosorb-3B (QuantaChrome, Graz, Austria). A degassing step at 200 °C for 20 h was conducted to remove surface adsorbates from the samples prior to analysis. Adsorption curves of the obtained isotherms were fitted via 7 points (relative pressures of 0.05–0.3) with the Brunauer-Emmett-Teller (BET) equation [26] to determine their specific surface area. Pore size distribution of the samples was evaluated by BJH method on the desorption part of the isotherms [27].

## 3. Results and Discussion

**Figure 2:**
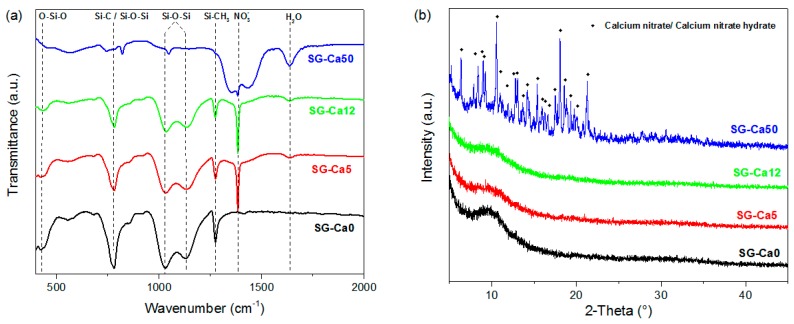
(**a**) FTIR spectra and (**b**) XRD diffractograms of xerogels SG-Ca0, SG-Ca5, SG-Ca12, and SG-Ca50.

The xerogels prepared as described above were investigated by means of FTIR spectroscopy. As evident from Figure 2a, the spectra of all prepared xerogels exhibit O–Si–O, Si–C, Si–O–Si, Si–CH_3_ vibrations [28,29,30]. Moreover, the Ca-containing xerogels, SG-Ca5, SG-Ca12, and SG-Ca50, show the presence of NO_3_^−^ vibration (ca. 1380 cm^−1^) and OH/H_2_O vibration (ca. 1630 cm^−1^) indicating the presence of residual nitrate and hydroxy groups after drying [22,23]. The peak intensity ratio of v(NO_3_^−^)/v(Si–O–Si) is shown to increase from SG-Ca5 to SG-Ca50, correlating to the increasing calcium nitrate loading from SG-Ca5 to SG-Ca50. The XRD patterns of the prepared xerogels (Figure 2b) indicate that SG-Ca0, SG-Ca5, and SG-Ca12 are x-ray amorphous; whereas crystalline calcium nitrate (hydrate) phases were observed in SG-Ca50. As reported in various case studies (e.g., Lin et al. [31]), calcium nitrate is still dissolved in pore liquids after gelation occurs during the sol-gel processing; thus, at this stage calcium nitrate is not incorporated into the sol-gel silicon-oxygen network. Consequently, the thermal treatment used to dry the gels resulted in the case of SG-Ca50 in the precipitation of crystalline calcium nitrate within the pores. This has not been observed for SG-Ca5 and SG-Ca12, most probably due to the significantly lower loading of calcium nitrate (Ca/Si = 0.05 and 0.12).

**Figure 3:**
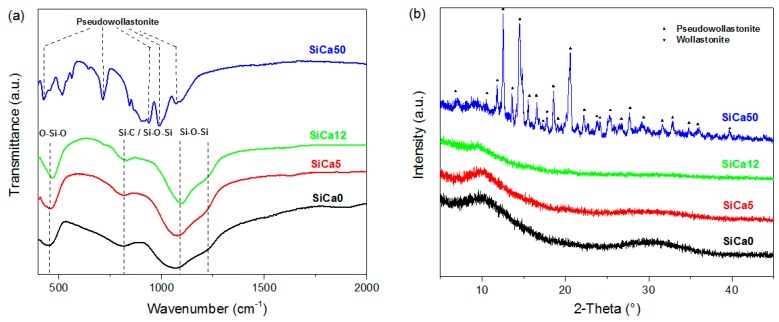
(**a**) FTIR spectra and (**b**) XRD diffractograms of SiCa0, SiCa5, SiCa12, and SiCa50 (samples obtained from corresponding xerogel precursors via pyrolysis at 1100 °C in Ar atmosphere).

The xerogels were converted thermally into silicon oxycarbide-based materials via pyrolysis in inert gas atmosphere. In Figure 3a, the FTIR spectra indicate that the methyl groups were completely removed after pyrolysis. Typical O–Si–O, Si–C and Si–O–Si vibration bands were identified for SiCa0, SiCa5, and SiCa12 [12]; whereas in the case of SiCa50 the observed bands were mainly assigned to calcium silicate (pseudowollastonite) [32]. The crystallization of pseudowollastonite along with small amounts of wollastonite in SiCa50 was confirmed by XRD analysis (Figure 3b). The crystallization of calcium silicates in SiCa50 was probably induced by the high amount of segregated calcium nitrate in the xerogel, which reacted with the silicon-oxygen network to form crystalline calcium silicate during the pyrolysis.

**Figure 4:**
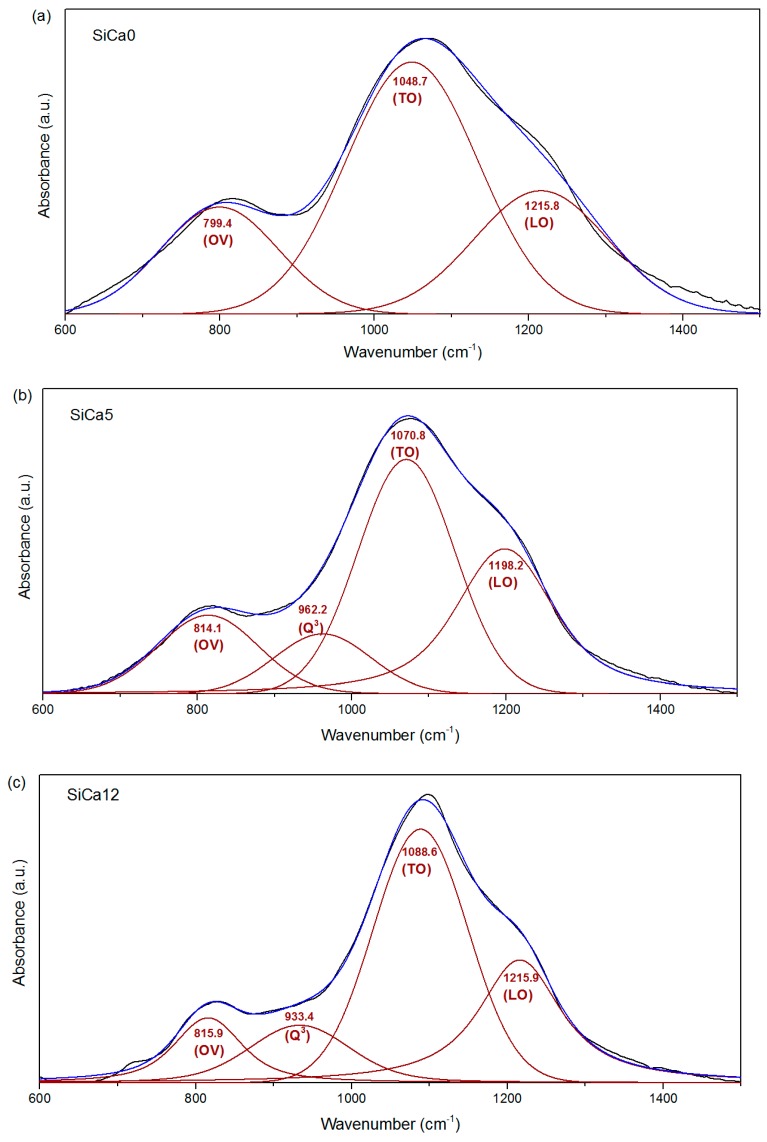
Deconvolution of FTIR spectra (black lines) between 600 cm^−1^ and 1500 cm^−1^ for (**a**) SiCa0, (**b**) SiCa5, and (**c**) SiCa12 into LO ν(Si–O–Si), TO v(Si–O–Si), overlapping v(Si–O–Si)/v(Si–C) (OV) and Q^3^ components (red lines). The sum of all fitted bands yields the blue lines in spectra.

For the amorphous oxycarbides SiCa0, SiCa5, and SiCa12, FTIR spectroscopic data were used in order to assess their network architecture. As shown in Figure 4, four vibration bands have been fitted by Voigt functions: (i) Band centered at 1190–1220 cm^−1^, corresponding to the longitudinal-optic (LO) Si–O–Si asymmetric stretching [33,34]; (ii) band centered at 1040–1090 cm^−1^, corresponding to the transverse-optic (TO) Si–O–Si asymmetric stretching [33,34]; (iii) band centered at 800–815 cm^−1^, corresponding to the overlapping (denoted as OV) of symmetric Si–O–Si stretching, bending Si–O–Si vibration, and Si–C vibration [30,33,34,35]; (iv) band centered at 900–970 cm^−1^, corresponding to SiO_4_ tetrahedral units containing one non-bridging-oxygen (Q^3^) [35,36]. As shown in Figure 4, the Q^3^ component is found only in the fitted spectra of Ca-containing silicon oxycarbides, i.e., SiCa5 and SiCa12, clearly demonstrating the effect of Ca as network modifier in the silicon oxycarbide network. This is in agreement with ^29^Si NMR data reported on Ca-containing silicon oxycarbides prepared from a Ca-acetylacetonate-modified polysilsesquioxane [12].

Since the band OV at ~800 cm^−1^ comprises both contributions from Si–O–Si and Si–C vibrations, the Q^3^/OV area ratio can be used to estimate the fraction of Q^3^ sites among the total Si species contained in the silicon oxycarbide network. As shown in Table 2, a significant increase of Q^3^/OV ratio from SiCa5 to SiCa12 is observed, corresponding to the increased Ca amount incorporated in the network of SiCa12 (as compared to that of SiCa5). Additionally, since Si–C vibration only appears in the OV band, the OV/(LO + TO) area ratio may be used to assess the fraction of Si species with Si–C bond in the silicon oxycarbide network. Interestingly, the OV/(LO + TO) ratio shows a decreasing tendency from SiCa0 to SiCa12, implying the decrease of network carbon content with increasing Ca^2+^ incorporation in amorphous network. This was reported in previous studies related to the modification of silicon oxycarbide networks with earth alkaline network modifiers [7,12].

Fidalgo et al. [33] ascribed the LO-TO splitting in silicate glass networks to long-range coulomb interactions, and found out that a reduced LO-TO splitting may be related to the presence of high porosity, which disturbs the long-range coulomb interaction. As shown in Table 2, SiCa5 and SiCa12 show much lower LO-TO splitting than SiCa0, thus it is concluded that the prepared Ca-containing silicon oxycarbides possess significantly larger porosity than that of the ternary silicon oxycarbide SiCa0.

The elemental compositions of the silicon oxycarbide samples prepared in this study can be estimated from their C and O contents and by assuming that Ca is present as calcium silicate in SiCa50 and as network modifier at the Q^3^ sites in SiCa5 and SiCa12 (see Table 3). All prepared silicon oxycarbide samples are shown to contain certain amount of segregated carbon, so-called “free carbon” [37]. Moreover, the decrease of the content of network carbon with increasing Ca^2+^ incorporation, which was concluded based on the evaluation of the OV/(LO + TO) ratio in the FTIR spectra, is clearly confirmed. The decrease of the carbon content in the oxycarbide network as well as its slight depolymerization due to the network modifier effect of Ca are considered to lead to decreased network connectivity in the series from SiCa0, to SiCa5, and to SiCa12 [12].

**Figure 5:**
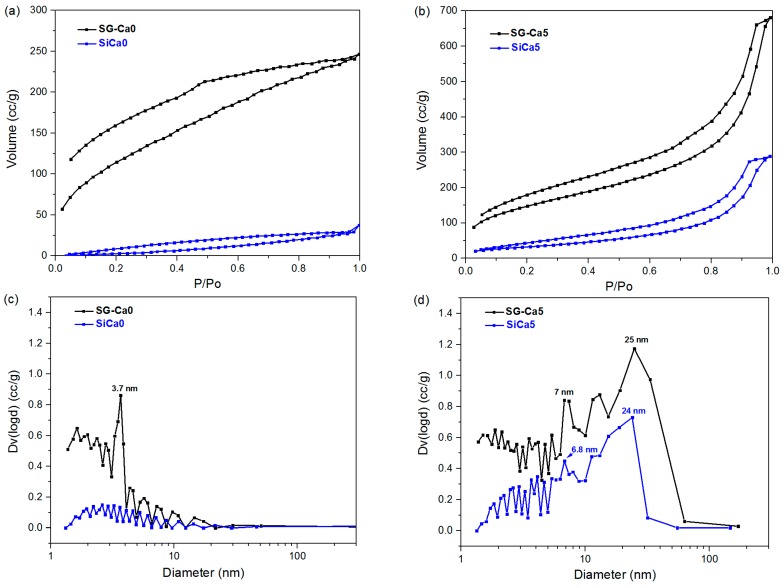
N_2_ sorption isotherms of SG-Ca0 and SiCa0 in (**a**), SG-Ca5 and SiCa5 in (**b**) and the BJH desorption particle size distribution calculated for SG-Ca0 and SiCa0 in (**c**) and SG-Ca5 and SiCa5 in (**d**).

**Figure 6:**
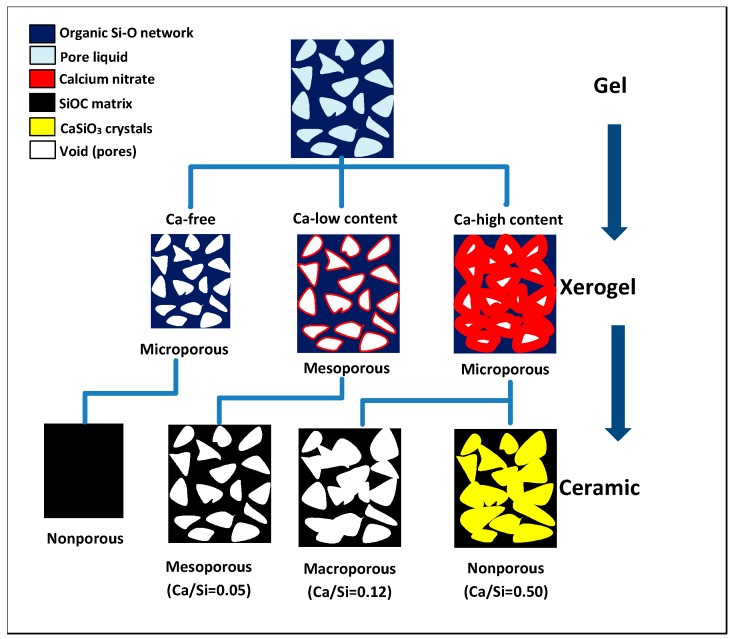
Schematic illustration of the porosity evolution from gels to xerogels, and then to silicon oxycarbide materials and the porosity dependency on the designed Ca/Si molar ratio.

The porosity of the xerogels as well as of the silicon oxycarbide samples was analyzed with N_2_ sorption. As shown in Figure 5, SG-Ca0 has an isotherm with mixed features of type I and type IV, while SG-Ca5 has a type IV isotherm [38]. BJH pore size distribution of SG-Ca0 confirms the presence of high content of micropores (< 2 nm), and also a substantial amount of mesopores at 3.7 nm (Figure 5c); whereas, SG-Ca5 contains a dominating part of mesopores around 25 nm (Figure 5d). Thus, SG-Ca0 shows significantly lower pore size as compared to SG-Ca5, which corresponds to the significant shrinkage observed during gel drying for SG-Ca0 compared to SG-Ca5 (Figure 1). Since capillary pressure induces pore shrinkage during drying [39], calcium nitrate precipitation from pore liquid (as mentioned before) during drying in SG-Ca5 may have probably stabilized pore walls, preserving the mesoporous gel structure in SG-Ca5 xerogel. On the other hand, although SG-Ca12 and SG-Ca50 are almost shrinkage-free (Figure 1), their isotherms are mainly microporous type I (see Figure S1 in support information), indicating a pore-filling effect of calcium nitrate. Thus, calcium nitrate loading shows two effects on the porosity of xerogels: (i) At a low amount (Ca/Si = 0.05), calcium nitrate precipitation during gel drying stabilizes mesopores; (ii) at high amounts (Ca/Si = 0.12 and 0.50), precipitated calcium nitrate fills up pores, reducing the pore size, as illustrated in Figure 6.

Interestingly, after pyrolysis at 1100 °C, the achieved SiCa5 has preserved its mesoporous structure (Figure 5d), while SiCa0 lost almost all of the micropores and mesopores observed in SG-Ca0 (Figure 5c). The collapse of micropores was also observed on SiCa12 and SiCa50 (see Figure S1 in support information). Since small pores contribute at the most to specific surface area [17], a significant decrease of BET specific surface area was observed for all samples after pyrolysis (Table 4). The collapse and closure of micropores during the polymer-to-ceramic transformation of PDC ceramics have been investigated in various studies and were found to occur mainly at relatively high temperatures (> 600 °C), where the gas evolution from thermal decomposition of residual organic groups ceased [17,40,41]. In contrary, mesopores have been found to be more resistant against temperature [14] and show only a slight decrease of pore size after pyrolysis in the present study (Figure 5d).

**Figure 7:**
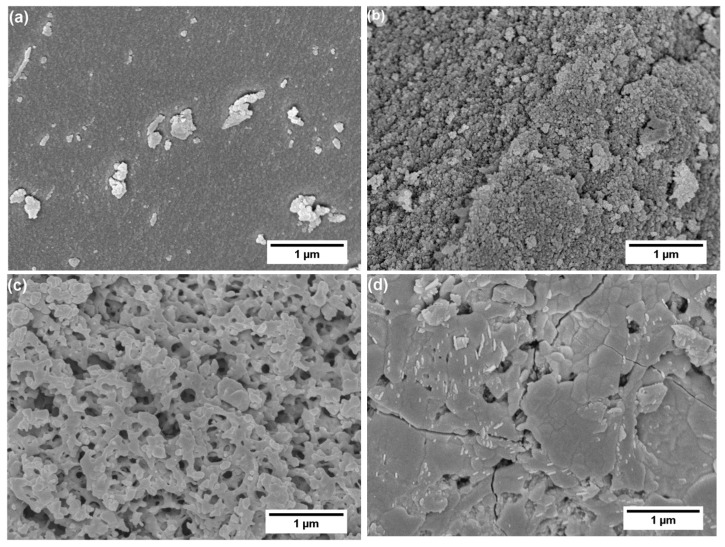
SEM surface morphology analysis of (**a**) SiCa0, (**b**) SiCa5, (**c**) SiCa12, and (**d**) SiCa50.

Furthermore, SEM surface morphology confirms the mesoporous structure of SiCa5 (Figure 7b), while SiCa12 shows macroporosity (Figure 7c). The observed high porosity of SiCa5 and SiCa12 shows a good agreement with their low LO-TO splitting (Table 2). Since SG-Ca12 is microporous, the macropores in SiCa12 must have been formed during the pyrolysis, namely via gas evolution during the removal of either residual organic groups or calcium nitrate, which decomposes at 500–700 °C by outgassing NO_2_, O_2_, and N_2_ [42]. At a relatively high amount (Ca/Si = 0.12), precipitated calcium nitrate could have reached certain crystal/cluster size in SG-Ca12. Compared to nonporous SiCa0 (Figure 7a), the decomposition of such crystals/clusters would leave voids, which are able to grow to macropores with ongoing gas evolution. On the other hand, SiCa50 is mainly nonporous with surface morphology similar to that of pseudowollastonite (Figure 7d) [43,44]. Pseudowollastonite has good sinterability at 1100 °C [45,46], and the sintering effect is considered to be responsible for the closure of pores left by calcium nitrate decomposition. Thus, calcium nitrate has different effects on the porosity of pyrolyzed silicon oxycarbide materials depending on its content: (i) At a low content (Ca/Si = 0.05), calcium nitrate decomposition does not influence the obtained mesoporosity noticeably; (ii) at a relatively high content (Ca/Si = 0.12), calcium nitrate decomposition induces the formation of macropores; (iii) at a significantly high content (Ca/Si = 0.50), calcium nitrate reacts with the Si–O gel network to form crystalline calcium silicate, which sinters at 1100 °C and thus induces the elimination of pores, as illustrated in Figure 6.

**Figure 8:**
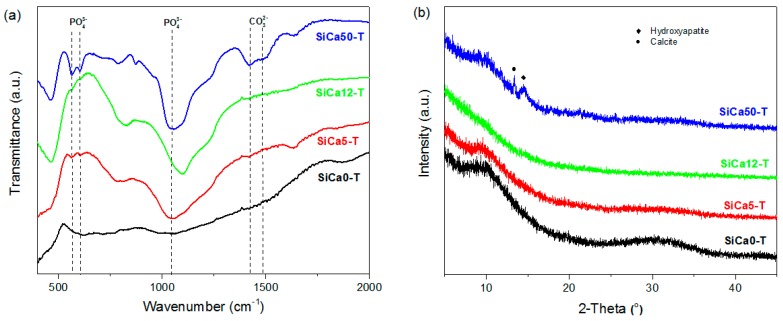
(**a**) FTIR spectra and (**b**) XRD diffractograms of silicon oxycarbide samples after seven days of SBF test.

In order to verify and compare the bioactivity of Ca-containing silicon oxycarbides, their apatite forming ability upon exposure to SBF solution was evaluated. FTIR spectra of SiCa5-T and SiCa50-T, i.e., SiCa5 and SiCa50 after seven days of SBF test, show typical PO_4_^3−^ vibrations at 565 cm^−1^, 605 cm^−1^, and 1047 cm^−1^ (see Figure 8a), referring to the formation of hydroxyapatite-like calcium phosphate [47,48,49]. In the case of SiCa12-T, only two small absorption notches at 565 cm^−1^, 605 cm^−1^ were observed, and the 1047 cm^−1^ vibration disappears under Si–O–Si vibration band. The precipitation of calcium phosphate on SiCa12-T is thus considered to be very limited. For SiCa0-T, no noticeable phosphate vibration is observable. XRD analysis (see Figure 8b) confirms besides the formation of crystalline hydroxyapatite also the formation of calcite (CaCO_3_) on SiCa50-T. It has been reported, that the formation of calcite is induced by a high concentration of Ca^2+^ in SBF solution [50]. By taking into consideration the high Ca/Si molar ratio (0.50) in SiCa50-T, which leads to the formation of dissolvable calcium silicate (pseudowollastonite) phase [51], a high Ca^2+^ release is expected to have induced the calcite formation. Additionally, SiCa5-T and SiCa12-T are shown to be x-ray amorphous. The FTIR detectable calcium phosphate on their surfaces is thus either not crystallized or the amount of crystallized hydroxyapatite is still not significant to be detected by XRD.

**Figure 9:**
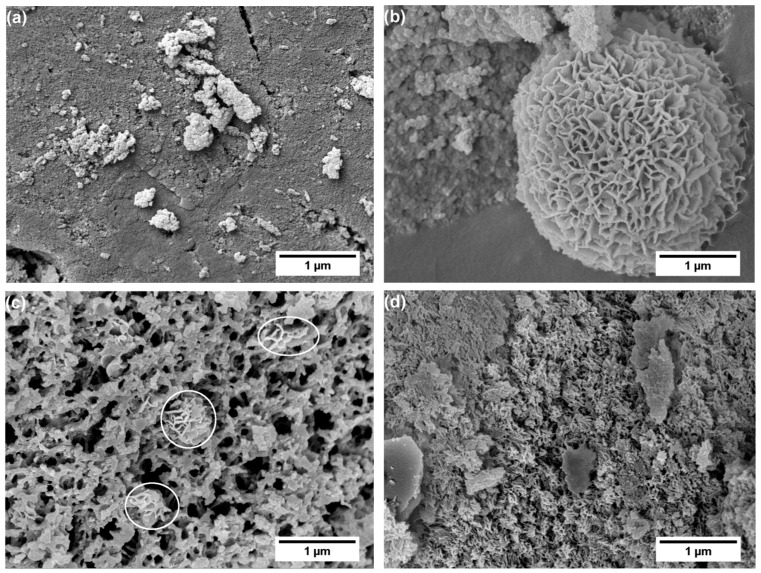
SEM surface morphology analysis of silicon oxycarbide samples after seven days SBF test: (**a**) SiCa0-T, (**b**) SiCa5-T, (**c**) SiCa12-T, and (**d**) SiCa50-T. The hydroxyapatite precipitation is marked in white circles for SiCa12-T.

SEM analysis of SiCa5-T, SiCa12-T, and SiCa50-T confirms the typical cauliflower-like morphology of hydroxyapatite on their surface (see Figure 9) [52,53]. Interestingly, although no hydroxyapatite-like precipitation was observed on SiCa0-T (Figure 9a), as also suggested by FTIR and XRD results, its surface shows a sort of coarsening compared to the surface before SBF soaking (see Figure 7a), indicating the occurrence of certain surface dissolution or leaching processes during SBF exposure. Indeed, Si release from ternary silicon oxycarbide takes place even though at a slow rate, according to our previous study [54]. The different shape and surface coverage of hydroxyapatite (HA) in Figure 9 shows clearly that SiCa50-T, with an almost complete HA surface covering, has the highest apatite forming ability among the investigated silicon oxycarbide samples.

A comparison between SiCa5-T and SiCa12-T shows more active apatite formation on SiCa5-T than on SiCa12-T: Hydroxyapatite grows on SiCa5-T to a size of several micrometers; whereas, hydroxyapatite clusters on SiCa12-T are much smaller (~0.5 um). Furthermore, the higher HA formation on SiCa5-T is also confirmed by FTIR results (see Figure 8a).

Therefore, although SiCa12 with higher Ca content (Q^3^ fraction) and lower network carbon (Table 2) is expected to have lower network connectivity than SiCa5 (as mentioned before), the apatite forming ability, i.e., bioactivity, is higher in SiCa5. It is well known, that low network connectivity leads to high dissolution of bioactive glasses and thus high bioactivity [55]. However, the bioactivity is also influenced/determined by surface kinetics, as sol-gel derived bioactive glasses with high specific surface area could extend the range of SiO_2_ content for showing bioactivity [56,57]. Thus, the observed discrepancy between network connectivity and apatite forming ability in the present study is probably influenced by the specific surface area in the same way. Mesoporosity in SiCa5 provides nearly three times higher specific surface area than that of macroporous SiCa12 (Table 4). Consequently, SiCa5 shows higher apatite forming ability.

Therefore, the introduction of mesoporosity to silicon oxycarbide proves to be an effective method to improve the apatite forming ability. Furthermore, mesoporosity is beneficial for obtaining additional functionalities, such as drug delivery ability or angiogenic effects. With limited Ca loading (Ca/Si = 0.05), SiCa5 is amorphous after 1100 °C pyrolysis. This high crystallization resistance is desirable if thermal processing of the material is anticipated.

## 4. Conclusion

Calcium nitrate-based sol-gel synthesis proves to be applicable for preparing Ca-containing silicon oxycarbide materials (SiCaOC). The porosity of synthesized SiCaOC is shown to be tunable depending on the content of calcium nitrate: (i) Calcium nitrate precipitation from pore liquids during gel drying is considered to induce both anti-shrinkage and pore-filling effects, resulting in mesoporous xerogel at a low content (Ca/Si = 0.05) and microporous xerogel at a high content (Ca/Si = 0.12 or 0.50); (ii) the decomposition of clustered calcium nitrate precipitated in xerogel during polymer-to-ceramic transformation is correlated to the formation of macropores at Ca/Si = 0.12, while higher loading with Ca/Si = 0.50 leads to the formation of crystalline calcium silicate (pseudowollastonite), the good sinterability of which leads to pore closure at pyrolysis temperature. The mesopores of xerogel at Ca/Si = 0.05 show a high resistance against shrinkage and collapse at pyrolysis temperature, leading to mesoporous SiCaOC.

SBF assessment confirms apatite forming ability of all SiCaOC materials. Besides the network depolymerization due to Ca incorporation at Q^3^ sites, high specific surface area in the case of mesoporous SiCaOC is shown to improve apatite forming ability significantly. Thus, the facile approach for preparing SiCaOC in the present study has achieved two bioactivity-enhancing effects simultaneously (i.e., Ca incorporation and the generation of mesoporosity). The combination of mesoporosity, crystallization resistance, bioactivity and biocompatibility makes the investigated mesoporous SiCaOC suitable for potential bone tissue engineering applications.

## Figures and Tables

**Table 1:** Designed molar ratios for xerogel samples and the corresponding amounts of chemicals applied for the sol-gel processing.

Sample	Molar Ratio (Ca/Si)	Triethoxymethylsilane (g)	0.01M HNO_3_ Solution (g)	Ca(NO_3_)_2_·4H_2_O (g)
SG-Ca0	0.00	10	3.0	0.00
SG-Ca5	0.05	10	3.5	0.66
SG-Ca12	0.12	10	4.0	1.59
SG-Ca50	0.50	10	4.5	6.62

**Table 2:** Percentual fractions of band area and area ratios of fitted bands from the FTIR spectra shown in Figure 4 and the calculated LO-TO splitting for SiCa0, SiCa5, and SiCa12.

Sample	LO Si–O–Si (%)	TO Si–O–Si (%)	OV Si–O–Si and Si–C (%)	Q^3^ (%)	Q^3^/OV	OV/(LO + TO)	LO-TO splitting (cm^−1^)
SiCa0	26.8	53.1	20.1	-	-	0.25	167.1
SiCa5	33.3	41.6	14.6	10.5	0.7	0.20	127.4
SiCa12	28.8	47.3	11.1	12.8	1.1	0.15	127.3

**Table 3:** Elemental contents, empirical formulae, and estimated phase compositions of the prepared silicon oxycarbide-based samples.

Sample	Si (wt.%)	O (wt.%)	C( wt.%)	Ca (wt.%)	Empirical Formulae	Estimated Phase Compositions
SiCa0	47.92^a^	40.18	11.90	-	Si_1_O_1.47_C_0.58_	Si_1_O_1.47_C_0.27_ + 0.31 C
SiCa5	44.49^a^	41.65	10.68	3.18^a^	Si_1_Ca_0.05_O_1.64_C_0.56_	Si_1_Ca_0.05_O_1.64_C_0.21_ + 0.35 C
SiCa12	40.93^a^	42.64	9.41	7.02^a^	Si_1_Ca_0.12_O_1.82_C_0.54_	Si_1_Ca_0.12_O_1.82_C_0.15_ + 0.39 C
SiCa50	30.79^a^	41.10	6.11	22.00^a^	Si_1_Ca_0.50_O_2.34_C_0.46_	0.50 Si_1_O_1.68_C_0.16_ + 0.50 CaSiO_3_ + 0.38 C

^a^: Si and Ca contents are calculated from the difference to 100 wt.% and the Ca/Si molar ratios given in Table 1.

**Table 4:** BET specific surface area (SSA) values calculated for xerogel samples and silicon oxycarbide samples from N_2_ sorption isotherms.

BET Specific Surface Area SSA (m^2^/g)	Ca/Si Molar Ratio = 0.00	Ca/Si Molar Ratio = 0.05	Ca/Si Molar Ratio = 0.12	Ca/Si Molar Ratio = 0.50
Xerogel	435.4	534.6	493.0	157.8
Silicon oxycarbide	22.5	123.4	39.5	23.9

## Supplementary Materials

The following are available online at https://www.mdpi.com/1996-1944/12/23/3862/s1, Figure S1: N_2_ sorption isotherms of SG-Ca12 and SiCa12 in (a), SG-Ca50 and SiCa50 in (b) and the BJH desorption particle size distribution calculated for SG-Ca12 and SiCa12 in (c) and SG-Ca50 and SiCa50 in (d).
