# Supplementary material for: Facile Preparative Access to Bioactive Silicon Oxycarbides with Tunable Porosity
Source: Materials (Basel). 2019 Nov 22;12(23):3862. doi: 10.3390/ma12233862 (PMC6926626; doi:10.3390/ma12233862)
Supplement: Supplementary file 1 [file materials-12-03862-s001.pdf]

Supplementary Materials: Facile Preparative Access to Bioactive Silicon Oxycarbides with Tunable Porosity

Fangtong Xie [1], Emanuel Ionescu [1,*], Marcela Arango-Ospina [2], Ralf Riedel [1], Aldo R. Boccaccini [2], Isabel Gonzalo-Juan [1]

[1] Institute of Materials Science, Technische Universität Darmstadt, Otto-Berndt-Str. 3, D-64287 Darmstadt, Germany; xie@materials.tu-darmstadt.de (F.T.X.); riedel@materials.tu-darmstadt.de (R.R.); gonzalo@materials.tu-darmstadt.de (I.G.-J.)

[2] Institute of Biomaterials, University of Erlangen-Nuremberg, Cauerstrasse 6, D-91058 Erlangen, Germany; marcela.arango@fau.de (M.A.-O.); aldo.boccaccini@ww.uni-erlangen.de (A.R.B.)

* Correspondence: ionescu@materials.tu-darmstadt.de

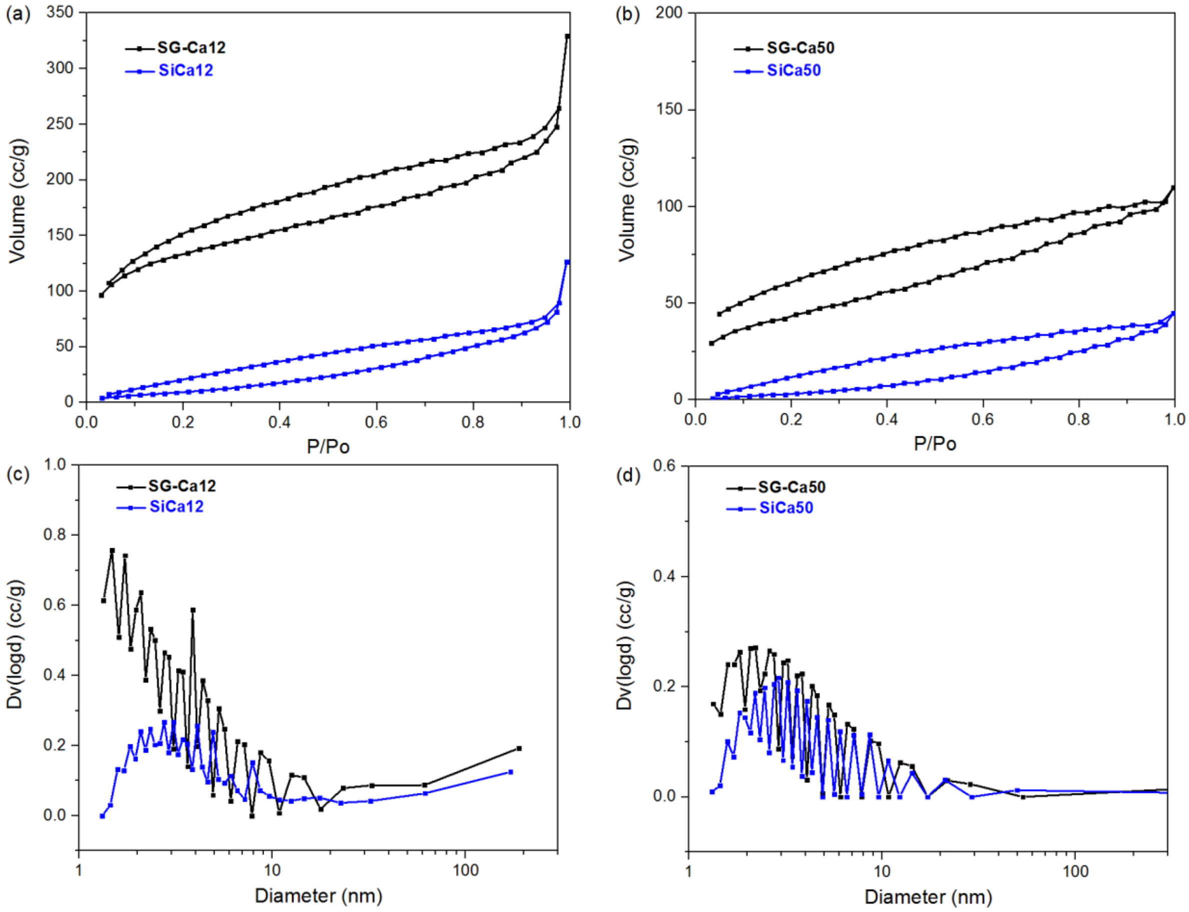

Figure S1. N_2 sorption isotherms of SG-Ca12 and SiCa12 in (**a**), SG-Ca50 and SiCa50 in (**b**) and the BJH desorption particle size distribution calculated for SG-Ca12 and SiCa12 in (**c**) and SG-Ca50 and SiCa50 in (**d**).
